# Supplementary material for: Are carbon emissions trading and green financial instruments synergistic? -Comprehensive quantitative research based on content analysis
Source: PLoS One. 2024 Mar 7;19(3):e0298601. doi: 10.1371/journal.pone.0298601 (PMC10919601; doi:10.1371/journal.pone.0298601)
Supplement: S1 Data — (ZIP) [file pone.0298601.s001.zip › Data/地级市Z_F工作报告.zip!/░▓╗╒/┴∙░▓╩╨/└┤╫╘╣1⁄2╓┌║┼í╛┬φ┐╦╩2╛▌═°í┐_2017.docx]

各位代表：

现在，我代表市人民政府，向大会报告政府工作，请予审议，并请市政协委员和其他列席人员提出意见。

一、砥砺奋进，良好开局的2016年

过去的2016年，是六安发展史上极不平凡的一年。这一年，我们倍感振奋，习近平总书记视察安徽第一站就来到六安，并作出重要指示，充分体现了对老区工作的高度重视、对老区人民的亲切关怀，为我们指明了前进方向、提供了根本遵循。这一年，我们也倍感压力，受宏观经济环境复杂多变、新旧动能转换曲折艰难等多重因素影响，年初我们遭遇了多年少有的经济下滑不利开局，稳增长、调结构、惠民生、防风险任务繁重而艰巨。一年来，在市委的坚强领导下，全市人民认真贯彻党的十八大和十八届三中、四中、五中、六中全会精神，以习近平总书记视察六安为动力，坚持红色土地绿色发展，坚持稳中求进工作总基调，创新发展理念，保持战略定力，团结拼搏，砥砺奋进，实现了经济运行企稳回升、趋势向好，促进了民生改善、社会和谐，取得了决战全面小康的良好开局。实现地区生产总值1108.1亿元、增长7.2%；完成财政收入153.3亿元、增长10%。

主要做了以下工作：

**第一，全力促进经济稳定增长。**全面贯彻落实中央、省稳增长各项政策措施，支持实体经济特别是小微企业发展，实现规模工业增加值381.2亿元、增长6.8%。深入开展“暖企行动”，市财政安排7亿元设立政府投资引导基金，兑现工业发展专项资金1.3亿元。成功举办市第一届工业品展销会。推动项目建设提质增效，完成固定资产投资1075亿元、增长8.2%。霍山应流航空和海洋装备产业园、六安火车站新站房、裕安大别山湿地公园、六安传化信实公路港等项目开工建设，金寨协鑫集成高效光伏组件生产、叶集中至信家居一期、舒城中鑫模具产业园一期、金安毛坦厂中学分校、霍邱体育中心等项目竣工运营。不断完善城乡流通体系，实现社会消费品零售总额541.5亿元、增长11.7%。扎实推进省级现代生态农业产业化示范市建设，实现农产品加工产值805.9亿元、增长3.5%；开展粮食绿色增产模式攻关，实现粮食总产314.2万吨。加快构建新型农业经营体系，新认证省级示范联合体13家、省级农业产业化龙头企业19家，西商食品新华畜牧被认定为农业产业化国家重点龙头企业，新培育农民合作社872家、家庭农场2911户。

**第二，扎实推进供给侧结构性改革。**积极稳妥去产能，因企施策处置“僵尸企业”，成功盘活企业300多家，清理闲置资产40多亿元、闲置土地1.2万亩；六大高耗能行业占工业总量比重下降至17.2%。中心城区工业企业“退城进园”工作稳步推进。供需发力去库存，销售商品房928.7万平方米、增长147.4%，总量、增幅分别居全省第2位、第1位，其中市区销售609万平方米、增长125.7%；商品住房库存面积减少368.1万平方米，去化周期由18个月降至10个月。分类施策去杠杆，新增政府债券41.54亿元，置换债券70.87亿元，节约政府债务利息3亿元。畅通金融渠道，金融机构贷款余额1192.8亿元、增长20.5%，新增贷款202.5亿元、增长85.8%；发放 “4321”新型政银担贷款51.4亿元、“税融通”贷款7.9亿元、创业担保贷款11.4亿元，使用续贷过桥资金21亿元。积极对接多层次资本市场，实现直接融资65.11亿元，上市报审企业1家，新增“新三板”挂牌企业6家、在审4家。合肥科技农商行六安分行获批筹建。多措并举降成本，严格实行涉企收费清单制度，累计减免缓抵各类税费31亿元。

**第三，深入实施绿色发展战略。**着力推进平台建设，合六叶工业走廊371家规模工业企业累计实现增加值133.5亿元，对全市规模工业增长贡献率达43.2%；完成“一谷一带”发展规划编制，累计实施项目306个，完成投资67.4亿元，成功举办第二届六安茶谷开茶节。深入实施创新驱动，新认定国家高新技术企业20家、国家级科技企业孵化器1家，实现高新技术产业增加值91.1亿元、增长8.2%。授权发明专利360件、增长40.6%，新增省级院士工作站5家。六安开发区、霍山开发区获批首批省级知识产权示范园区。大力发展战略性新兴产业，实现产值278.6亿元、增长12.9%，霍山高端装备基础零部件战新园区纳入省级战新基地，金寨先进光伏制造纳入省级重大试验工程，全市省级产业集群专业镇发展到20个、居全省第1位。大力发展现代服务业，服务业增加值占生产总值比重由36.2%提高至37.4%。继续实施旅游产业重点项目三年行动计划，接待游客3579.5万人次，实现旅游综合收入255.5亿元，分别增长22.3%、26%；荣获全国旅游“厕所革命”先进市，舒城万佛湖国家5A级旅游景区正式挂牌，霍山、金寨入围首批国家全域旅游示范区，金寨入选全国休闲农业和乡村旅游示范县。加强生态建设，实现节能减排年度控制目标，成功创建全国低碳试点城市；深入开展大气污染防治，全年空气优良率81.3%，六安城区PM10平均浓度下降18%，降幅居全省第1位；金寨获批全国首个国家高比例可再生能源示范县，霍山荣获全国养生宜居县，霍邱入围全国秸秆综合利用试点县，舒城通过国家生态县验收；扎实推进大别山区水环境生态补偿，全市集中饮用水源水质达标率、六大水库水质优良率、河流考核断面水质优良率均为100%，荣获全省水生态文明城市。全面完成森林增长工程。加快推进质量品牌升级工程，新增国家地理标志保护产品1个、地理标志证明商标8件、省著名商标38件、安徽名牌15个、农业“三品”认证产品25个、国家级“守合同重信用”公示企业7家，叶集入围全国产业集群区域品牌建设试点区，金寨成功创建国家级农产品质量安全县，霍山荣获国家出口农产品质量安全示范区。

**第四，继续深化改革开放。**完成叶集区行政区划调整，霍山撤县设市正式申报。深入推进“放管服”改革，市级权力事项精简率达54.8%，行政审批事项精简率达49.3%。完成市县不动产登记改革。市直生产经营服务类事业单位转企改制基本完成，机关事业单位养老保险制度改革顺利实施，机关公车改革全面完成。深化商事制度改革，全面实施“五证合一、一照一码”，新登记各类市场主体3.7万户。全面推开“营改增”试点，实现总体税负只减不增。全面完成农村集体土地确权登记颁证工作。启动农村“三变”改革试点。金寨国家级农村宅基地改革试点稳步实施，成功实现城乡建设用地增减挂钩指标跨市交易。深化农村金融综合改革，金寨国家农村信用体系建设试验区和全国“两权”抵押贷款试点扎实推进。深化医药卫生体制改革，推动公立医院综合改革，着力构建分级诊疗制度，推动医药、医疗、医保联动改革。深入开展公共资源交易管理体制改革，市县一体化交易平台整合基本完成，招投标领域专项整治取得阶段性成果。持续扩大对外开放，成功举办中国·六安对外合作恳谈会、六安与央企深化合作对接会、六安“走进中关村”恳谈会、德国六安文化周等系列推介活动；强化产业链招商、专题招商，实现到位内资445亿元、增长11.3%，利用外资3.8亿美元、增长10.3%，华夏幸福杭埠产业新城、安徽弘泰高分子材料、大别山四季彩色生态观光园等项目成功落户。加快区域融合发展，皖江示范区、大别山革命老区振兴、皖北结对合作等区域发展政策进一步落实，合肥都市圈基础设施互联互通、环境污染联防联控、产业发展分工协作、市场体系统一构建等取得积极成果。

**第五，统筹新型城镇化发展。**结合行政区划调整，启动新一轮城市总体规划修编，完善控制性详细规划和各类专业规划。积极开展“多规合一”、 海绵城市、智慧城市等专项试点。巩固深化省级文明城市创建成果，深入开展“五城同创”，成功创建国家森林城市、国家节水型城市。加快中心城区重点工程建设，共实施项目66项、完成政府性投资39亿元。大力推进征迁工作，中心城区重点工程完成征迁168.8万平方米。深入推进城镇规划建设管理提升行动，金寨新县城、霍山县城、叶集新城区建设步伐加快，舒城、霍邱县城规划建设中等城市力度加大，霍山成功创建国家园林城市。特色小集镇建设全面启动，集镇建成区整治深入推进，裕安区独山镇荣膺首批中国特色小镇。持续推进美丽乡村建设，第三批68个省级中心村建设全面完成，金寨、裕安、舒城荣获全省美丽乡村建设先进县。加快现代综合交通体系建设，完成道路建设投资75亿元，建成国省干线公路236公里，大别山旅游扶贫快速通道建成通车；合安高铁舒城段开工建设，德上高速六安段、合阜高速霍邱段等项目前期工作有序推进。淠河、史河治理工程基本完成，灾后水毁水利工程修复与防洪薄弱环节建设性治理扎实推进，治理水土流失面积132.8平方公里。严格耕地资源保护，开展永久基本农田划定，补充耕地1.5万亩，建设高标准基本农田160万亩。

**第六，持续保障和改善民生。**城镇、农村常住居民人均可支配收入分别达到24728元、9960元，均增长8.3%。大力实施民生工程，投入各类资金117亿元。中心城区公共自行车一期项目运营良好，公共停车场项目启动建设。市区公交体制改革顺利完成，公交保障水平明显提升，荣获首批创建省优先发展公共交通示范城市。城镇新增就业6.1万人，城镇登记失业率4%。社会保障制度实现全覆盖，在全省率先整合新农合和城镇居民医保，建立统筹城乡的医保体系，医保参保率达99%以上，五保、低保和社会救助制度不断完善。新开工各类保障性安居工程32851套，改造农村危房14320户。有效应对6·30洪涝灾害，重建因灾倒损房屋 4637间。市残疾人康复中心建成使用。继续实施学前教育第二个三年行动计划、全面改善农村义务教育薄弱学校五年规划，启动六安城区义务教育阶段公办学校第二个三年行动计划，推进新一轮普通高中布局调整，金寨、舒城顺利通过全国义务教育发展基本均衡县评估验收。加快职业教育发展，推动中高职有效衔接，金寨职业学校获批设立技师学院。健全城乡公共文化基础设施，市图书档案馆、科技文化馆主体工程竣工，新建农民文化乐园57个。打造“社区一家亲、文化进万家”等群众文化活动品牌。组队参加全省“中华经典诵读”比赛荣获第1名。广泛开展全民健身运动，成功举办市第四届运动会。有序实施全面两孩政策，提高生育服务管理水平，人口自然增长率8‰。

**第七，切实维护社会和谐稳定。**健全应急管理体制机制，做好防灾减灾工作，应对突发事件的预防预警和处置能力有效提升。扎实推进安全生产监管体系建设，安全生产形势总体平稳。深入开展农产品、食品、药品安全专项整治，保障人民群众饮食用药安全。着力推进农村平安服务网建设，加快建设立体化、数字化社会治安防控体系，严厉打击各类违法犯罪活动，扎实开展特殊人群管理，人民群众安全感、满意度不断提升。启动“七五”普法，加强法律援助和社区矫正安置帮教工作。深入推进信访制度改革，信访秩序有所好转。加强国防动员、民兵预备役工作，深入开展双拥共建，军民融合深度发展，第三次荣获全国双拥模范城。广播电视、新闻出版、统计、审计、档案、地方志、外事侨务、民族宗教、老龄、妇女儿童、社会慈善等事业得到加强，气象、地震、人民防空等工作取得新进展。

各位代表！2016年是决战全面小康的开局之年，习近平总书记视察金寨时强调：“全面建成小康社会，一个不能少，特别是不能忘了老区”。一年来，我们认真贯彻落实习近平总书记重要指示，紧紧围绕精准扶贫精准脱贫，建立 “1+N”政策体系，加大资金投入，市县财政预算安排扶贫资金23.7亿元，预计全年减少贫困人口11.5万人，出列贫困村25个，贫困发生率降至6.8%以下。全面实施脱贫攻坚十大工程，实施产业扶贫工程，帮扶近4万贫困户发展特色种养业，建设村、户光伏电站11891个，建成农村电子商务服务站点近3000个，申报省级乡村旅游扶贫重点村60个；实施就业扶贫工程，建立完善就业脱贫和转移就业信息台账，新增农村劳动力转移就业6.2万人，推荐贫困人口到就业扶贫基地和用人单位创业、就业2.7万人；实施智力扶贫工程，对1.6万名贫困农民进行实用技术培训和专业技能培训，发放贫困家庭子女就学补助资金1.38亿元；实施健康扶贫工程，为11.8万患有慢性病、4.8万患有大病的贫困人口建立健康扶贫档案，落实贫困人口医疗费用“351”兜底保障政策；实施社保兜底扶贫工程，农村低保标准由1944元/人/年上调至3000元/人/年，达到国家扶贫线标准的95%以上；实施易地扶贫搬迁工程，完成7561名贫困人口搬迁任务；实施基础设施建设扶贫工程，完成小型农田水利改造提升工程投资3.45亿元，解决17.2万人安全饮水问题，完工农村道路畅通工程4353公里，完成农村电网改造投资2.18亿元；实施生态保护扶贫工程，发放各类公益林补偿资金8546万元，完成85个村环境综合整治；实施金融扶贫工程，发放扶贫再贷款3亿元、扶贫小额贷款1.24亿元；实施社会扶贫工程，扎实开展扶贫日活动，认领项目资金1.49亿元，积极推进民营企业“百企帮百村”精准扶贫行动。完善各级党委、政府及部门脱贫攻坚责任体系，健全“单位包村、干部包户”和驻村扶贫工作队管理制度，建立严格的考核和问责机制。

各位代表！过去的一年，全市政府系统始终把学习贯彻习近平总书记视察安徽、视察六安重要讲话精神作为重大政治任务，扎实开展“两学一做”学习教育，积极开展“讲看齐、见行动”学习讨论，不断提升政府工作水平。严格落实政府决策机制，切实执行政府决策程序。规范执法行为，畅通行政复议和行政调解渠道。加强政府权力运行监督，深化政务公开，实行“互联网+政务服务”，权责清单全面进入平台运行。进一步加强机关效能建设，全面实施“三查三单”制度，政府工作执行力、落实力得到提升。自觉接受人大依法监督、政协民主监督和社会舆论监督，共办理人大议案2件、代表建议111件和政协委员提案295件，办复率100%。加强党风廉政建设，推进审计监督全覆盖，开展酒桌办公等专项整治和“四风”突出问题“回头看”活动，大力推进督查、巡视、“电视问政”等发现问题的督促整改，严肃查处了一批违法违纪案件。

各位代表！ 过去的一年，困难和挑战前所未有，成绩和荣誉来之不易。这是省委、省政府和市委科学决策、正确领导的结果，是市人大、市政协和社会各界有效监督、大力支持的结果，是全市上下凝心聚力、顽强拼搏的结果。在此，我代表市人民政府，向大家表示诚挚的感谢！

在肯定成绩的同时，我们清醒地认识到，经济社会发展还存在不少困难和问题。主要是：经济下行压力加大，规模工业增加值、固定资产投资等指标增幅没有达到预期目标；结构性矛盾凸显，农业大而不强、工业主导地位不突出、服务业层次偏低的状况没有根本改变；实体经济困难依然较多，民间投资和制造业投资意愿不高、后劲不足；脱贫攻坚任务艰巨，教育、医疗、文化、社会保障等公共服务水平不高；政府工作执行力、管理服务效能仍存在薄弱环节，少数干部不敢为、不想为、不会为的现象不同程度存在；“四风”问题禁而不绝，顶风违纪问题时有发生，侵害群众利益的不正之风和腐败问题依然突出。对此，我们必须坚持问题导向，着力加以解决。

二、攻坚克难，扎实做好2017年工作

2017年是党的十九大召开之年，是实施“十三五”规划的重要一年，也是本届政府的收官之年，做好今年工作意义重大，影响深远。今年政府工作的总体要求是：全面贯彻党的十八大、十八届三中、四中、五中、六中全会精神和习近平总书记系列重要讲话特别是视察安徽重要讲话精神，认真落实省第十次党代会和市第四次党代会部署，围绕“打好五大战役、构建五大高地”奋斗目标，坚持稳中求进工作总基调，以绿色发展为总取向，以推进供给侧结构性改革为主线，以提高发展质量和效益为中心，以实施五大发展行动计划为总抓手，统筹做好稳增长、促改革、调结构、惠民生、防风险各项工作，促进经济持续健康发展和社会和谐稳定。

今年主要预期目标是：地区生产总值增长7.5%左右，财政收入增长8.5%，固定资产投资增长10%，规模以上工业增加值增长7.5%，社会消费品零售总额增长11.5%，到位境内资金和外商直接投资增长10%，外贸进出口总额持平，城镇、农村常住居民人均可支配收入分别增长8%和9%，新增城镇就业2.7万人，城镇登记失业率控制在4.5%以内，完成节能减排目标任务。

为此，重点抓好以下八个方面的工作：

**（一）加强供给侧结构性改革，夯实经济稳定增长根基。**

**着力振兴实体经济。**大力发展装备制造、绿色食品加工、新能源、生物医药、电子信息、采矿冶金及新材料等六大主导产业，力争主导产业增加值占全部工业增加值比重达到65%。制定实施“中国制造2025”六安篇，力争装备制造业首位度达到20%以上。加快星瑞齿轮三期、迎驾野岭产业园、中财管道二期、胜利电子二期、沃特玛产业园等项目建设，积极推动应流航空产业园、华润啤酒罐装线、为民新能源汽车电池等项目开工建设。继续实施技术改造专项行动，力争投资增长10%以上，占工业投资比重达到60%。推进信息化和工业化深度融合发展，加快两化融合管理体系标准普及推广，争创省级数字化车间、智能工厂，培育省、市级两化融合示范企业15户以上。进一步清理和规范涉企收费，千方百计降低企业生产经营成本和制度性交易成本。继续开展“暖企行动”，扩大市工业发展专项资金、产业引导基金规模，帮助企业拓展地产品销售。

**保持有效投资力度。**深入实施“四督四保”制度，着力加大有效投入，依托国家重大建设项目库，动态调整充实三年滚动项目库，进一步加强政府投资项目储备，持续加大中央预算内资金、专项建设基金和地方政府债券争取力度。加快推进合肥至六安区域快线、金寨支线机场等项目前期工作，开工建设悠然南山二期、华电舒城舒茶风电场、温氏集团一体化养殖及加工等重点项目，加快金寨抽水蓄能电站、霍山大别山国际旅游度假区、吉宝皖江国际冷链物流园、紫荆花生态养老服务中心、叶集双渡电商物流园等项目建设，完成亿元以上项目投资680亿元。推进政府与社会资本合作，引导民间资本参与“三重一创”和国资国企改革。

**拓宽金融渠道。**持续深化新型政银担合作，拓宽“税融通”业务覆盖面，提升续贷过桥资金周转效率，力争新增贷款150亿元。创新间接融资方式，积极发展多业态金融机构，争取更多域外金融机构落户六安。增加市融资担保公司注册资本金2亿元。支持农村商业银行、村镇银行通过增资扩股或直接融资等途径补充资本，增强服务实力。强化企业上市挂牌推进机制，争取新增上市报备企业2家、“新三板”挂牌企业6家。推动企业通过股票和债券融资，力争直接融资65亿元以上。防范金融风险，持续优化金融生态环境。

**促进消费升级。**扩大大众旅游、养老健康和信息消费，增加中高端教育、医疗、文化、体育、养老等服务供给，促进电子商务、智能家居等消费。优化消费环境，引导发展家用轿车、家用信息设备、耐用消费品等消费信贷，提高新能源汽车在公共交通中的比例。深入推进便民服务体系建设，加快中心城区农贸市场和大型商品交易市场建设改造，规范建设社区综合商业服务中心，着力打造15分钟便民商圈。规范出租车、网约车服务和管理。

**（二）深入实施“全创改”，加快调结构转方式促升级。**

**深入实施创新驱动战略。**以培育壮大龙头企业、增强创新能力为抓手，推进产业链招商，完善产业配套，推动战略性新兴产业加快向主导产业转变。设立市战新基地建设专项引导基金，集中力量加快霍山高端装备基础零部件产业集聚发展基地、金寨先进光伏制造重大试验工程建设；围绕新一代电子信息、生物医药、新材料、新能源、绿色食品加工等产业，培育一批市级战新产业集聚发展基地。着力提升企业自主创新能力，力争新认定国家高新技术企业20家以上，实现高新技术产业增加值90亿元以上。加快推进六安大学科技园、众创空间等创新平台建设，新增省级科技孵化器或众创空间2-3家。加大各类人才引进力度，力争新引进高层次科技人才团队1-2个、授权发明专利150件以上。大力引进天使、风险、股权投资等基金，构建覆盖企业全生命周期的金融支撑体系。继续实施质量品牌升级工程，培养优秀企业家群体，发扬“工匠精神”，打造一批质量品牌建设示范县和示范企业，争创六安瓜片茶产业、霍山石斛产业全国知名品牌示范区。

**加快绿色发展平台建设。**进一步优化城镇发展空间布局和形态，加强城市特色风貌设计，彰显历史文化，提升城市品位，扩大城市影响力。全面实施合六叶工业走廊暨金叶一体化产业发展规划，突出高端装备制造、绿色食品加工、新能源、电子信息、生物制品制造、家居制造等产业，打造一批百亿级产业集群，把六安开发区打造成绿色振兴核心增长极。认真落实六安茶谷和淠河生态经济带建设规划，全面推进六安茶谷沿线特色小镇、茶谷小站和茶谷小院建设，大力发展生态农业、绿色食品加工、中药材、乡村旅游等特色产业；加快淠河生态经济带水利交通基础设施建设，开展全流域治理，加快培育优质高效农业和特色旅游文化产业。

**大力发展现代服务业。**围绕旅游发展“345”布局，继续实施旅游产业重点项目三年行动计划，完善基础设施配套，提升景区景点品位。加快六安城市旅游目的地建设，启动白马尖-佛子岭5A景区和六安茶谷国家旅游度假区创建。修编全市红色旅游规划，推动金寨、裕安独山苏维埃城等红色教育基地建设。加强旅游产品开发和营销体系建设，推进旅游与文化、生态、健康等产业深度融合。进一步规范旅游市场秩序。实施大健康产业规划，大力发展健康食品、运动、养生、养老等产业。推动工业设计、科技服务、金融保险、现代物流等生产性服务业发展，培育省市级工业设计中心10个以上。推动六安电商创业园、六安邮政快递电商物流园、霍山电商产业园、金寨“互联网+”大厦、舒城徽网在线等电商园区标准化、规范化建设，支持淘宝“特色中国•六安馆”、“邮乐购”、“蜂狂购”、“三个农民”等本埠电商平台发展，大力推进“互联网+物流快递”，进一步完善末端配送体系，实现区域、城乡物流快递协调发展。

**（三）加快转变农业发展方式，提升现代农业发展水平。**

**推进农业结构调整。**加强耕地保护，深化粮食流通体制改革，保障粮食安全。推进农业供给侧结构性改革，扩大蔬菜、食用菌、花卉苗木、中药材等特色经济作物种植比重，增加优质安全和市场紧缺农产品供应。建设一批规模化、标准化生产基地，培育壮大一批特色产业集群。积极发展多功能大循环农业，加快发展休闲观光农业，打造一批特色农业小镇和“一村一品”示范村。

**大力发展现代生态农业。**全面实施农业产业化提升行动，力争实现农产品加工业产值850亿元以上。积极培育现代农业产业化联合体，扶持发展土地股份、农村社区、联合社等新型合作组织，新增亿元以上龙头企业10家、农民合作社800家、家庭农场1000家。深入开展农业社会化服务“111”示范创建活动。组织实施新型职业农民培育工程，培训新型职业农民4000人。推进“互联网+农业”，促进一二三产融合发展。健全农产品质量安全体系，深入实施化肥农药使用量零增长行动，扩大农产品质量安全可追溯试点。建立监测预警和应急处置机制，加强重大动植物疫病防控。深入推进“三园两场”创建，实现规模种养基地生产过程标准化、规范化。支持 “三品一标”认证，力争新认证农产品20个以上。

**夯实农业发展基础。**认真实施农业综合开发等土地整理项目，实现田、林、路、渠、电等设施综合配套。实施农村道路畅通工程2837公里，推进“四好”农村路建设，加快农村公路建管养运一体化发展。加强淮河流域综合治理，推进防洪薄弱环节建设性治理，全面推行“河长制”，提升防洪保安、水资源供给、水生态修复能力。推进农村电网升级改造，加快信息网络建设。扎实开展第三次全国农业普查工作。

**（四）推进重点领域改革，进一步激发市场活力。**

**继续推进“放管服”改革。**完善权责清单动态调整机制，建立公共服务清单和中介服务清单制度，基本形成“3+2”清单制度体系。继续精简市级审批事项，清理和简化前置审批条件，规范和改进行政审批管理。加强政府权力运行事中事后监管，建立规范高效的监管机制。创新政府管理模式，推广“互联网+政务服务”。深化商事制度改革，进一步放宽市场准入管制。

**大力推进财税金融改革。**继续完善政府预算体系，进一步加大政府性基金预算、国有资本经营预算与一般公共预算的统筹力度。推进市以下财政事权和支出责任划分改革及税收征管体制改革。加大政府存量资产、财政存量资金、企业存量资源盘活力度，深化国资国企改革。深化政府性债务管理制度改革，建立规范的债务管理及风险预警机制。深化农村金融综合改革，探索开展新型农村金融组织发展试点，扩大农村土地承包经营权等“三权”抵押贷款，完善农村政策性融资担保体系和风险补偿机制。强化农村信用体系建设，进一步改善农村基础金融服务环境。

**深化农村综合改革。**全面落实农业支持保护政策，促进农民持续增收。积极利用农村土地确权登记颁证成果，开展农村土地“三权”分置改革试点。扩大集体资产股份合作和农村土地入股试点范围，开展农村“三变”改革试点。深入推进金寨国家农村宅基地制度改革试点，同步开展土地征收、集体经营性建设用地入市改革试点。鼓励开展特色农产品和设施农业保险。完成国有林场改革任务，进一步深化集体林权制度配套改革，引导和规范林权流转。完善重要农产品储备管理体制，支持符合条件的民营企业参与政策性粮食收储。

**（五）积极扩大开放合作，大力发展开放型经济。**

**提高招商引资质量和水平。**突出产业链招商，围绕核心企业，通过建链、补链、延链、强链，促进产业集聚。开展大健康产业、战新基地、大学生创业创新、特色小镇等专题招商。强化以商招商，积极寻求合作招商。积极通过实施产业引导基金、社会贡献考评奖励、综合环境保障等规范性政策措施，引导和支持境内外资金投向主导产业、战略性新兴产业和现代服务业。充分利用国家证监会支持贫困地区发展IPO绿色通道政策，切实抢抓机遇，加大工作力度，开展精准招商。

**推进区域合作。**推动合肥都市圈一体化发展，加快合六工业走廊产业发展、基础设施建设、环境保护等重大专题合作，争取合肥帮扶我市共建市级现代产业园，推动合肥高新区与霍邱县、包河区与舒城县合作园区建设，支持蜀山区和金安区战略合作。认真落实《大别山革命老区振兴发展规划》，强化交通建设、旅游开发等领域合作。

**提升经济外向度。**抢抓“一带一路”机遇，强化政策支持，促进外贸、外资、外经联动发展。促进传统优势外贸产业做大做强，支持外贸企业参加境内外展会，扩大机电、汽车零部件、农产品等产品出口。积极孵化外贸企业，加快引进一批潜力大、发展好、带动力强的外向型企业，培育外贸竞争新优势。积极发展进口贸易，支持企业扩大先进技术、设备和关键零部件进口。加快保税物流中心、公共保税仓库申报建设进度，促进贸易便利化。争取海关在六安设立正式机构。

**（六）加快新型城镇化建设，推进城乡区域协调发展。**

**大力推进农民工市民化。**深化户籍制度改革，建立城乡统一的户口登记制度，拓宽居住证的服务保障功能。落实进城落户农民土地承包经营权、宅基地使用权、集体资产收益分配权“三权”维护和自愿有偿退出办法。加快推进基本公共服务均等化，积极开发适合农业转移人口的就业岗位，力争进城农民进得来、稳得住、能致富。

**做大做强中心城市。**统筹推进东部产业新城、西部生态新区、绿色城南、叶集新城建设，进一步完善城市功能，提升综合承载力，彰显城市特色。继续实施中心城区重点工程，完成政府性投资50亿元。全面规范集体土地和国有土地上房屋征收工作。加强公园与绿地、道路与广场海绵体建设，提升防洪防涝能力。建立扁平化指挥、智能化监控、网格化管理、点对点调度的城市管理新模式，全面推进联勤综合执法。推进市政设施标准化建设，引入“互联网+大数据”，实现基础设施监测和公共服务智能化。以文明城市创建为总抓手，深入开展市容市貌、道路交通秩序整治，建立健全长效机制。加大棚改货币化安置力度，有序安排土地供应，推动商业用房转型利用，规范房地产开发、销售、中介行为和物业管理服务，促进房地产业持续健康发展。

**加快县城和城镇发展。**积极推进“多规合一”，支持霍山、舒城等创造条件争取县改市。加快城镇管理体制改革，理顺规划建设管理体制。继续推进“三治三增三提升”行动，加快通信网络、路网、管网、电网等基础设施建设。完善教育、文化、医疗等公共服务，加大县城棚户区改造力度，着力改善人居环境。加快重点镇建设，支持独山镇等特色小城镇建设，着力打造一批省市级特色小城镇。深入推进公共交通优先发展示范市建设。认真实施国省干线公路建设第二个三年行动计划，新改建国省干线公路148公里。

**深入推进美丽乡村建设。**修订完善美丽乡村建设规划，进一步优化空间布局。加快乡镇政府驻地整治建设，持续推进中心村建设及自然村环境整治，采用政府购买服务等方式，大力引进市场主体，实现农村垃圾污水处理社会化、专业化、长效化。实施“一村一品”、 “一乡一业”，推进发展特色产业，形成品牌效应。推进农民文化乐园建设，传承和发展农村文化，提升农民文化素养。

**（七）加大扶贫攻坚力度，着力保障改善民生。**

**坚决打赢脱贫攻坚年度战役。**建立全覆盖的精准扶贫、监测评估、防范返贫、责任落实四项机制，完成12.755万人脱贫、228个贫困村出列任务。深入实施脱贫攻坚十大工程，大力发展特色种养、光伏、电子商务、乡村旅游等产业，积极探索产业扶贫实现路径，着力构建“新型经营主体+贫困户”模式；实现贫困家庭“雨露计划”和建档立卡贫困户学生资助全覆盖；全面落实健康扶贫政策，有效减少因病致贫、因病返贫；完善兜底扶贫措施，实现农村低保线和扶贫标准线“两线合一”；深入实施易地扶贫搬迁、基础设施建设、生态保护扶贫工程，进一步改善贫困人口生产生活条件；健全小额扶贫贷款风险补偿机制，扩大贷款覆盖面。推进“百企帮百村”精准扶贫行动，开展扶贫日活动和扶贫志愿者行动。完善脱贫攻坚责任体系，强化责任落实，严格责任考核。充实和加强基层扶贫队伍，夯实各项工作基础。

**巩固提升民生工程。**在认真实施省33项民生工程基础上，继续实施市区城乡公交一体化、中心城区公共自行车服务系统、城区停车场建设3项市级项目。完善多元投入机制，鼓励引导社会、企业和个人投资民生工程。强化民生工程建设质量监管，加大后期管养经费投入，健全长效管养机制。建立市级民生工程项目进退机制。加大民生工程审计监督力度，严格绩效考评。引进社会资本参与公共文化设施建设运营。

**做好就业和社会保障工作。**深入实施各类创业计划，加强高校毕业生、返乡农民工、退役士兵等群体的创业服务，以创业带动就业。全面落实各项社会保障和社会救助政策，编密织牢社会保障安全网，突出抓好困难群众基本生活保障。扎实推进城乡医保统筹，全面实施全民参保登记计划。逐步提高企业退休职工基础养老金、优抚对象抚恤和生活补助、城乡低保对象补助水平。扩大住房公积金保障范围。扎实做好农民工工作，全面治理拖欠农民工工资问题。稳步推进机关事业单位养老保险制度改革。

**加强环境综合治理。**以生态环保重大工程建设和生态文明示范创建为重要抓手，加快完善生态环境治理体系。继续推进总量减排，持续加强污染防治，加快环境监测、环境监察达标能力建设。实施“三尘、三气”等大气污染专项整治，加强重点流域、区域水环境综合治理。启动实施第二次全市污染源普查。强力推进各县区生态文明示范创建工作，争创国家环保模范城市。启动实施林业增绿增效行动，推动新一轮退耕还林工程，继续强化森林防火和森林有害生物防治。

**（八）创新社会治理，维护社会和谐稳定。**

**统筹发展社会事业。**着力培育和践行社会主义核心价值观，大力开展群众性精神文明创建活动。完善公共文化体育服务体系，确保市图书档案馆、科技文化馆投入运营，开工建设市体育中心、市广播电视塔。加强文化遗产保护与利用，推进优秀传统文化传承保护。加大历史文化名城、名镇、名村申报与保护力度，认真开展乡镇志、名村志编纂工作。加快文体产业发展，积极争创全国文化产业示范园区和文化体育产业示范基地。统筹推进医药卫生体制改革，深化公立医院综合改革，全面推进县域医共体、市域医联体建设和家庭医生签约服务，促进分级诊疗。加强基层医疗卫生机构服务能力建设，努力构建和谐医患关系。启动学前教育第三个三年行动计划，抓好市区公办幼儿园建设。加快实施全面改善农村义务教育薄弱学校五年规划，扎实推进六安城区义务教育阶段公办学校第二个三年行动计划，确保裕安、霍邱（叶集）顺利通过全国义务教育发展基本均衡县评估验收。继续推进普通高中布局调整。加快职业教育和高等教育发展。深入贯彻全民健身国家战略，推进群众体育与竞技体育全面发展。改革完善计划生育服务管理，进一步强化出生人口性别比综合治理。认真做好统计、物价、气象、地震、对台、档案、邮政、外事侨务、老龄、妇女儿童、残疾人等工作。

**提升社会治理水平。**深入推进“七五”普法，加强社区矫正监管，推进人民调解化解矛盾研判预警机制建设，扩大法律援助。严厉打击各类违法犯罪活动，加强治安防控体系建设，深入推进反邪教斗争，深化“平安六安”建设。全面落实党的民族宗教政策，依法加强民族宗教事务管理。加强社区依法自治和协商民主制度建设，推进农村社区建设试点。坚持问题导向，以法治思维、责任落实、公开透明推动信访工作。完成全国第二次地名普查。

**强化公共安全管理。**健全灾害应急救助体系，加强防灾减灾救灾体制机制建设，深入开展全国综合减灾示范区和减灾示范县（区）创建工作。严格食品药品安全监管，开展省级食品药品安全城市创建工作，切实落实属地监管责任，强化社会共治格局。严厉打击各种扰乱市场经济秩序的不正当行为，维护公平有序的市场环境。严格落实安全生产责任制，加强重点行业领域事故防控，深入开展“铸安”行动，有效预防和遏制较大以上事故发生。加强国防动员、国防教育、兵员征集和人民防空工作，支持军队改革，巩固双拥创建成果，推进军民深度融合。

三、依法行政，着力提升政府工作水平

推动新常态下新发展，对政府工作提出了更高要求。我们要以新发展理念统领发展全局，不断加强自身建设，全面提升政府工作水平，努力向全市人民交上一份合格答卷。

**强化政治定力，始终做到忠诚看齐。**牢固树立“四个意识”，特别是核心意识、看齐意识，坚定不移向党中央看齐、向党的理论和路线方针政策看齐、向党中央决策部署看齐，坚决维护党中央权威，坚决维护习近平总书记党的领导核心地位，始终在思想上政治上行动上与党中央保持高度一致，结合实际创造性工作，确保中央和省、市委各项决策部署精准落实。坚定理想信念，强化理论武装，增强中国特色社会主义道路自信、理论自信、制度自信、文化自信，内化于心、外化于行，化为推动发展、造福人民的强大动力。

**强化法治思维，推进法治政府建设。**严格依法决策，落实重大行政决策合法性审查制度、终身责任追究制度，加强新型智库建设，发挥决策咨询、法律顾问等制度作用，运用大数据等手段提升决策水平。加强和规范干部教育培训，提升干部能力素质。认真落实行政执法责任制，规范执法行为。推进行政调解与人民调解、司法调解相衔接，完善行政复议制度，自觉接受司法监督。全面推进政务公开、办事公开，让权力在阳光下运行。自觉接受人大法律监督、工作监督和政协民主监督，认真办理人大代表议案、建议和政协委员提案，广泛听取各民主党派、工商联、无党派人士意见，充分发挥工会、共青团、妇联等人民团体重要作用。

**强化服务创新，提升政务服务效能。**改进政府服务方式，勇于破除思维惯性和路径依赖，善于以新理念、新方法指导实践。创新联系群众方法，坚持领导干部调查研究、定期接待群众来访、群众满意度测评等制度，多渠道了解社情民意、回应群众关切。树立崇尚实干、鼓励创新的工作导向，建立健全奖惩激励机制和容错纠错机制，着力解决不想为、不敢为、不会为问题。完善抓落实的制度体系，健全全面覆盖的督查机制，打通政策落实“中梗阻”和“最后一公里”，确保政府工作掷地有声、落地见效。持续深化机关效能建设，加大考核督查问责力度，严肃查处敷衍塞责、推诿扯皮等懒政怠政行为，不断改进政府机关作风。

**强化正风肃纪，加强反腐倡廉建设。**严格落实全面从严治党主体责任，巩固“两学一做”学习教育成果，持续提升政府系统从严管党治党水平。全面落实党风廉政建设责任制，认真履行“一岗双责”、“一案双查”制度。强化行政监察，健全完善公共资源交易监管制度，规范公共资源交易行为，加大公共资金、国有资产、自然资源等重点领域审计监督力度。严格落实中央八项规定、国务院“约法三章”和省、市委各项规定，严肃查处发生在群众身边的“四风”和腐败问题。健全廉政风险防控机制，注重家庭、家教、家风建设，着力塑造风清气正的政治生态。

各位代表！踏上新征程使命光荣，实现新发展任务艰巨。让我们紧密团结在以习近平同志为核心的党中央周围，在市委的坚强领导下，凝心聚力，奋发图强，为建设幸福六安、全面建成小康社会而努力奋斗，以优异成绩迎接党的十九大胜利召开！
